# Case Report: Discordant hypertrophic cardiomyopathy in monozygotic twins with LEOPARD syndrome and an identical PTPN11 c.1391G > C (p.Gly464Ala) mutation

**DOI:** 10.3389/fcvm.2026.1978684

**Published:** 2026-10-14

**Authors:** Xin Xue, Yayuan Ning, Fang Yao, Xiaowei Zhang, Xianjing Song

**Affiliations:** Department of Cardiology, Second Hospital of Jilin University, Changchun, Jilin, China

**Keywords:** hypertrophic cardiomyopathy, left ventricular outflow tract obstruction, LEOPARD syndrome, monozygotic twins, noonan syndrome with multiple lentigines, PTPN11, RASopathy

## Abstract

LEOPARD syndrome (LS; Noonan syndrome with multiple lentigines) is a rare autosomal dominant RASopathy in which PTPN11 mutations cause hypertrophic cardiomyopathy (HCM) with highly variable expressivity. We report adolescent monozygotic twin brothers carrying an identical PTPN11 c.1391G > C (p.Gly464Ala) mutation that arose *de novo*, who developed discordant cardiac phenotypes despite sharing a genome and an intrauterine and postnatal environment. The younger twin developed obstructive HCM with systolic anterior motion of the mitral valve and left ventricular outflow tract obstruction (peak resting gradient 36 mmHg, peak Valsalva gradient 61 mmHg), together with pectus excavatum and macroglossia; the older twin had non-obstructive HCM (maximal septal thickness 12 mm) with a patent foramen ovale. Both twins had multiple lentigines, facial dysmorphism, and sensorineural hearing loss requiring cochlear implantation. The younger twin was treated with a beta-blocker and activity restriction, whereas the older twin was managed with surveillance alone. This case demonstrates that an identical RASopathy mutation can produce divergent forms of HCM, supports a role for non-genetic modifiers in cardiovascular expression, and underscores the need for hemodynamics-guided, individualized cardiovascular management in LS.

## Introduction

LEOPARD syndrome (LS; OMIM #151100), also known as Noonan syndrome with multiple lentigines, is a rare autosomal dominant RASopathy (1–3). Cardiovascular involvement, particularly hypertrophic cardiomyopathy (HCM), is the major determinant of morbidity and mortality in LS and affects approximately 75% of patients (4). Compared with sarcomeric HCM, LS-associated HCM tends to present earlier and to follow a more aggressive course (4, 5). Approximately 90% of cases are caused by missense mutations in PTPN11, which encodes the SHP-2 protein tyrosine phosphatase (1). In contrast to the gain-of-function PTPN11 mutations that cause Noonan syndrome, LS-associated mutations impair SHP-2 catalytic activity (6). LS shows complete penetrance but highly variable expressivity, and the basis of this variability, particularly for the cardiovascular phenotype, is poorly understood. We describe monozygotic twin brothers with an identical PTPN11 c.1391G > C (p.Gly464Ala) mutation who developed discordant HCM (obstructive versus non-obstructive), an observation that provides a rare opportunity to examine the non-genetic determinants of cardiac disease expression.

## Case description

### Patient information and presentation

Two adolescent monozygotic twin boys of Chinese Han descent were referred to our cardiology department for routine cardiac evaluation. They were born at 36 weeks of gestation by vaginal delivery after an uncomplicated pregnancy, and there was no family history of sudden cardiac death, congenital heart disease, or neurocutaneous disorders. The younger twin (Twin 2) had respiratory distress and cyanosis at birth and required neonatal intensive care; neonatal echocardiography showed hypertrophic obstructive cardiomyopathy. The older twin (Twin 1) was admitted to the neonatal unit for prematurity but had no respiratory distress or cyanosis. At approximately 2 years of age, both twins were found to have bilateral severe-to-profound sensorineural hearing loss (7) and underwent right-sided cochlear implantation, with near-normal auditory function afterward.

### Clinical findings at presentation

Multiple hyperpigmented macules appeared on the face, trunk, and extremities of both twins during early childhood. At presentation, both had height about 2 cm below the age-matched mean and showed mild ocular hypertelorism, low-set posteriorly rotated ears with prominent earlobes, and a broad forehead with a tapering chin. Multiple brown-black lentigines (2–5 mm) were distributed over the face, neck, trunk, and extremities (Figures 1A–C). Twin 1 had a normal chest wall, no cardiac murmur, and a normal tongue. Twin 2 had pectus excavatum, a 3/6 holosystolic murmur at the mitral area, a 3/6 systolic ejection murmur at the left upper sternal border, and macroglossia (Figures 1D,E). Both were normotensive.

**Figure 1:**
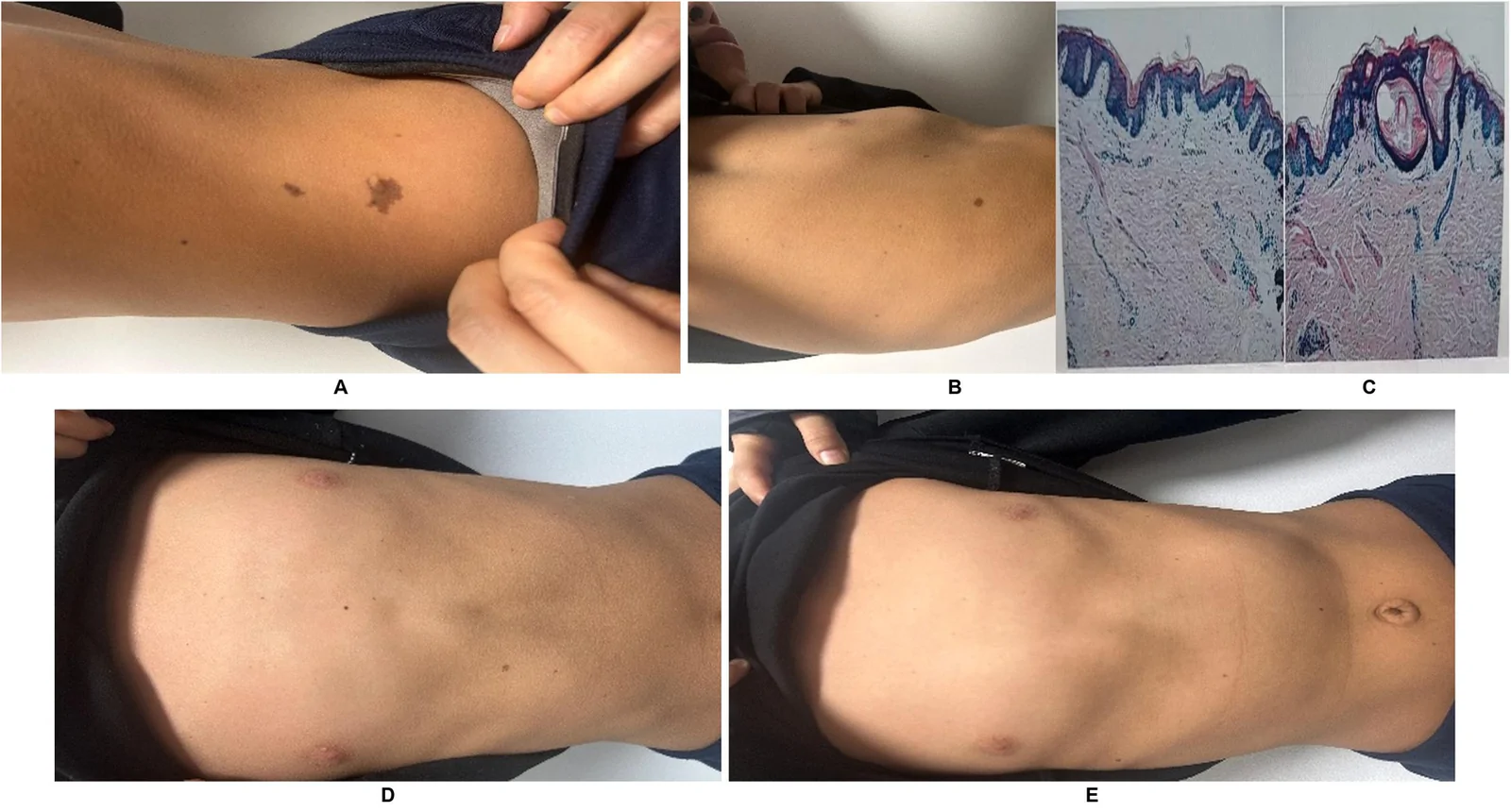
Clinical features. **(A)** Multiple lentigines on the trunk. **(B)** Multiple lentigines on the face. **(C)** Histopathology showing lentigo simplex. **(D)** Twin 1 with a normal chest configuration. **(E)** Twin 2 with asymmetric pectus excavatum.

### Diagnostic assessment

Electrocardiography showed normal sinus rhythm in Twin 1 and sinus rhythm with left ventricular high voltage in Twin 2 (Figures 2A,B). Transthoracic echocardiography (Figures 2C–F) showed asymmetric septal hypertrophy in Twin 1 (maximal septal thickness 12 mm), a non-dilated left ventricle, normal systolic function (left ventricular ejection fraction [LVEF] > 55%), no left ventricular outflow tract obstruction at rest or with the Valsalva maneuver, and a small patent foramen ovale. Twin 2 had more severe asymmetric septal hypertrophy, with a maximal left ventricular lateral wall thickness of 21 mm, a thickened basal interventricular septum (approximately 16 mm), systolic anterior motion of the mitral valve, and left ventricular outflow tract obstruction with a peak resting gradient of 36 mmHg and a peak Valsalva gradient of 61 mmHg; moderate mitral regurgitation was present and LVEF >70%. Cardiac magnetic resonance imaging could not be performed because of the cochlear implants. Next-generation sequencing of a RASopathy gene panel identified a heterozygous PTPN11 c.1391G > C variant (NM_002834.5) in exon 12, resulting in a p.Gly464Ala substitution in the PTP domain of SHP-2, in both twins (Figure 3). Sanger sequencing confirmed that neither parent carried the variant, indicating that it arose *de novo*. No variants were detected in RAF1, BRAF, MAP2K1, MAP2K2, KRAS, NRAS, HRAS, SHOC2, CBL, or SOS1. Both twins met the diagnostic criteria for LS (8).

**Figure 2:**
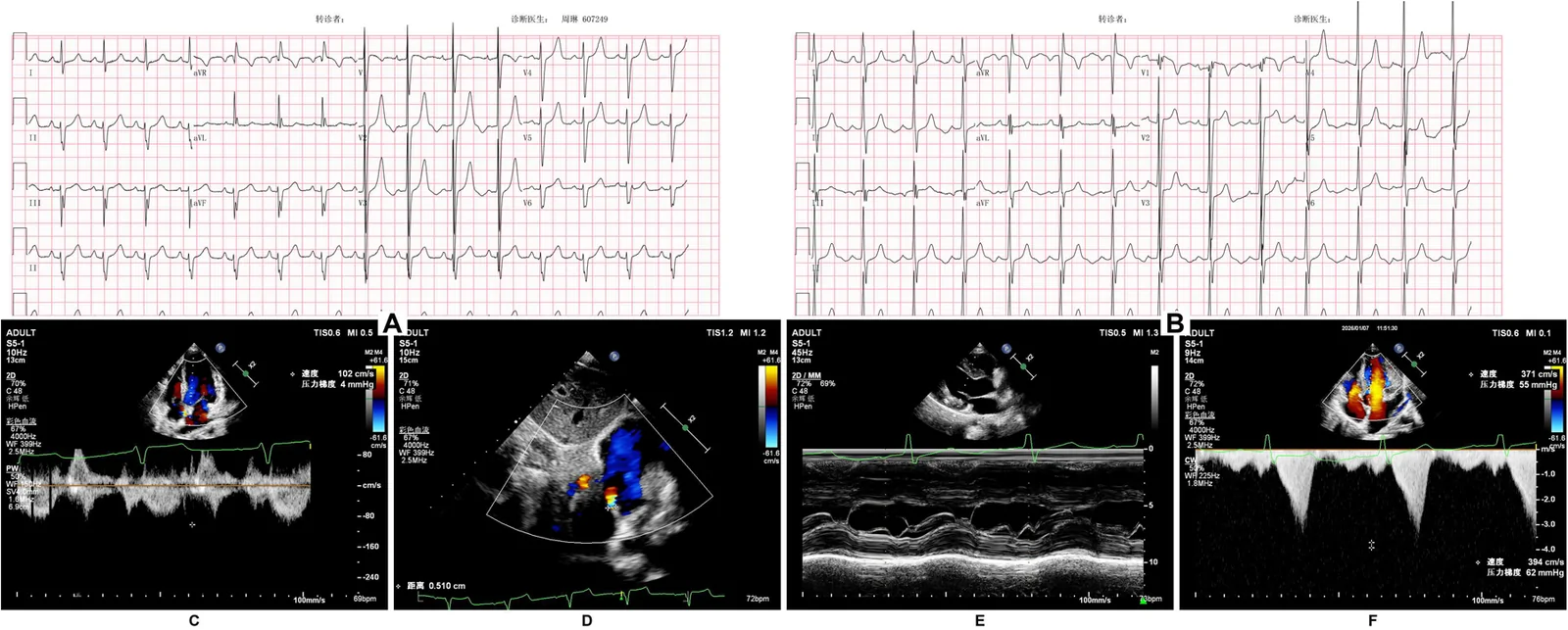
Cardiac investigations. **(A)** Electrocardiogram of Twin 1 showing normal sinus rhythm. **(B)** Electrocardiogram of Twin 2 showing left ventricular high voltage. **(C,D)** Echocardiography of Twin 1 showing asymmetric septal hypertrophy **(C)** and a patent foramen ovale **(D) (E,F)** Echocardiography of Twin 2 showing systolic anterior motion of the mitral valve **(E)** and left ventricular outflow tract obstruction **(F).**

**Figure 3:**
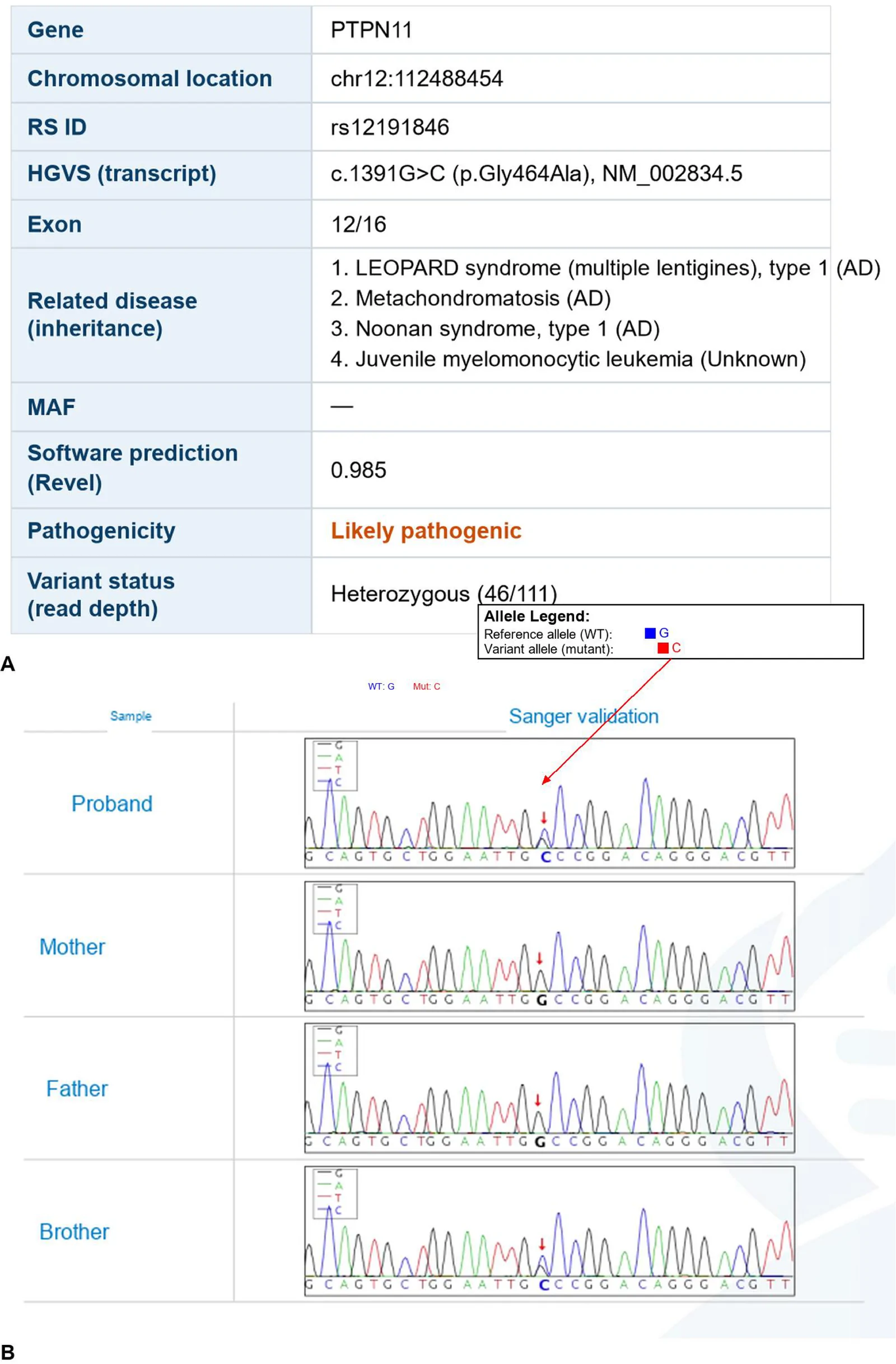
Genetic analysis. **(A)** Variant interpretation for the PTPN11 c.1391G > C (p.Gly464Ala) variant, including gene, chromosomal location, RS ID, HGVS notation, exon, related diseases, minor allele frequency, software prediction (REVEL score), pathogenicity classification, and variant status with read depth. **(B)** Sanger sequencing chromatograms confirming the heterozygous c.1391G > C variant in both twins (Proband and Twin 1) and wild-type sequence in both parents (Mother and Father), indicating a *de novo* mutation. Red arrows indicate the variant position.

### Therapeutic intervention and follow-up

Twin 2 began metoprolol succinate 23.75 mg once daily for symptomatic left ventricular outflow tract obstruction (9) and was advised to avoid competitive sports and strenuous activity. Twin 1 was managed with regular surveillance. Both twins are followed annually with electrocardiography and echocardiography, and the family received genetic counseling (Table 1).

**Table 1:** Timeline of the episode of care.

Age/time	Clinical event
Birth (36 weeks gestation)	Twin 2: respiratory distress, cyanosis, neonatal hypertrophic obstructive cardiomyopathy. Twin 1: prematurity without distress
Early childhood (approximately 2 years)	Both twins: bilateral severe-to-profound sensorineural hearing loss; right-sided cochlear implantation
Childhood	Both twins: progressive lentigines on the face, trunk, and extremities. Twin 1: skin biopsy showing lentigo simplex
Adolescence (at presentation)	Both twins: cardiac evaluation. Twin 1: non-obstructive HCM with patent foramen ovale. Twin 2: obstructive HCM with left ventricular outflow tract obstruction (36 mmHg), pectus excavatum, macroglossia. Genetic testing: PTPN11 c.1391G > C (p.Gly464Ala), *de novo*
Management	Twin 2: metoprolol 23.75 mg once daily, activity restriction. Twin 1: surveillance
Ongoing	Annual electrocardiography and echocardiography

## Discussion

The p.Gly464Ala variant is a rare but recurrent LS-associated mutation in the PTP domain of SHP-2; functional studies show that it impairs catalytic activity, consistent with the loss-of-function mechanism of LS-associated PTPN11 mutations (6, 10). It has been reported in both familial and sporadic cases of LS with variable expressivity (11, 12).

The most instructive feature of this twin pair is the discordant cardiac phenotype. Twin 2 developed severe obstructive HCM with left ventricular outflow tract obstruction, chest deformity, and macroglossia, whereas Twin 1 had non-obstructive hypertrophy with a patent foramen ovale. Because monozygotic twins share a genome and, in this case, a common intrauterine and postnatal environment, this discordance points to non-genetic factors in cardiac disease expression, including stochastic variation in RAS-MAPK signaling during cardiac development, differences in intrauterine hemodynamics, or somatic mosaicism in cardiac modifier genes (13, 14). The macroglossia in Twin 2 is unusual for LS (15) and may reflect more pronounced RAS-MAPK-mediated soft-tissue overgrowth, paralleling his more severe cardiac phenotype.

This case has practical implications for cardiovascular management. First, the severity of the phenotype at presentation did not predict the clinical course, supporting individualized, lifelong cardiac surveillance in all patients with LS. Second, the discordant phenotypes suggest that decisions about targeted therapy, such as the MEK inhibitors under evaluation for RASopathy-associated HCM, should be guided by each patient's current hemodynamic status (the presence and severity of outflow tract obstruction) rather than by genotype alone (16). Limitations include the inability to perform cardiac magnetic resonance imaging because of the cochlear implants, and the lack of functional or epigenetic studies in our patients.

## Patient perspective

As this condition is caused by a genetic mutation, there is currently no targeted treatment available. Current management primarily involves beta-blockers and symptomatic therapy, which cannot fundamentally improve prognosis, particularly for symptoms arising from outflow tract obstruction and heart failure with preserved ejection fraction. Both children currently use cochlear implants; although their hearing is reduced compared to normal individuals, they can generally maintain normal daily activities and attend school. Since the underlying pathological cardiac remodeling and disease progression cannot be halted, there is a substantial long-term risk of myocardial fibrosis and sudden cardiac death. Furthermore, whether this disease-causing gene will be inherited by future generations remains a significant concern for our family.

## Data Availability

The original contributions presented in the study are included in the article. Further inquiries can be directed to the corresponding author.
