# Supplementary material for: Case Report: Discordant hypertrophic cardiomyopathy in monozygotic twins with LEOPARD syndrome and an identical PTPN11 c.1391G > C (p.Gly464Ala) mutation
Source: Front Cardiovasc Med. 2026 Oct 14;13:1978684. doi: 10.3389/fcvm.2026.1978684 (PMC13713445; doi:10.3389/fcvm.2026.1978684)
Supplement: Supplementary file 1 [file Datasheet1.docx]

**Supplementary Material**

*Manuscript: Case Report: Discordant Hypertrophic Cardiomyopathy in Monozygotic Twins with LEOPARD Syndrome and an Identical PTPN11 c.1391G>C (p.Gly464Ala) Mutation*

**Supplementary Table S1. Clinical features of LEOPARD syndrome: literature comparison with the present case.**

| **Clinical Feature** | **Reported Prevalence** | **Twin 1** | **Twin 2** |
| --- | --- | --- | --- |
| Multiple lentigines | ~85% | + | + |
| Café-au-lait spots | ~50% | + | + |
| Hypertrophic cardiomyopathy | ~75% | + (non-obstructive) | + (obstructive) |
| LV outflow tract obstruction | ~34% of HCM | - | + |
| PFO/ASD | ~10-15% | + (PFO) | - |
| ECG abnormalities | ~75% | - | + (LVH voltage) |
| Pulmonary stenosis | 10-25% | - | - |
| Ocular hypertelorism | ~70% | + | + |
| Low-set ears | ~60% | + | + |
| Sensorineural hearing loss | 15-25% | + (cochlear implant) | + (cochlear implant) |
| Growth retardation | <50% | Mild | Mild |
| Pectus deformity | NR | - | + (excavatum) |
| Macroglossia | NR | - | + |
| Cryptorchidism | ~30% | - | - |
| Intellectual disability | ~10-20% | - | - |
| PTPN11 mutation | ~90% | c.1391G>C (p.Gly464Ala) | c.1391G>C (p.Gly464Ala) |

LV, left ventricular; LVOTO, left ventricular outflow tract obstruction; PFO, patent foramen ovale; ASD, atrial septal defect; ECG, electrocardiogram; NR, not reported.

**Supplementary Table S2. Differential diagnosis of LEOPARD syndrome vs. other RASopathies.**

| **Feature** | **LEOPARD Syndrome** | **Noonan Syndrome** | **CFC Syndrome** | **NF1** |
| --- | --- | --- | --- | --- |
| Lentigines | +++ (hallmark) | +/- (rare) | +/- | +/- (CALMs) |
| HCM | +++ (~75%) | + (~20%) | + (~40%) | - |
| Pulmonary stenosis | + (10-25%) | ++ (~50%) | + | - |
| Intellectual disability | Mild (~10-20%) | Mild-moderate | Moderate-severe | Variable |
| PTPN11 mutation type | LOF (phosphatase decreased) | GOF (phosphatase increased) | N/A | N/A |
| RAS-MAPK pathway | Decreased activity (context-dependent) | Increased activity | Increased activity | Increased activity |
| Malignancy risk | Low | Moderate | Low | High |

CFC, cardiofaciocutaneous; NF1, neurofibromatosis type 1; HCM, hypertrophic cardiomyopathy; LOF, loss-of-function; GOF, gain-of-function; CALMs, café-au-lait macules.
